# Highlight: The “Out of Pollen” Hypothesis—In Plants, Animals New Genes Originate in Male Sex Tissue

**DOI:** 10.1093/gbe/evu224

**Published:** 2014-10-27

**Authors:** Danielle Venton

Evolution is the story of shifting genes: New ones arise, old ones pass away, existing ones become more or less widespread in response to the pressures of life. Without the creation of new genes this dance would not be possible. New research suggests that, regarding new gene creation, plants and animals have much more in common than meets the eye.

More than a decade ago research groups began to publish papers detailing the evolutionary patterns of new genes in animals, noting that they often seemed to arise in male sex organs. This work developed and became known, eventually, as the “out of testis” hypothesis, where the testis, the male reproductive organ, is the origin for the birth and evolution of new genes (Kaessmann 2010). But now geneticists from the Chinese Academy of Sciences and the University of Toronto have proposed an “out of pollen” hypothesis, showing that in plants too, new genes seem to arise in the male sex cells. Their research was recently published in *Genome Biology Evolution* (Wu et al. 2014).

“This inclusion of the pollen case makes the whole picture bizarre and even more interesting,” says Manyuan Long from the Department of Ecology and Evolution at the University of Chicago.

Long did some of the original work which laid the basis for the “out of testis” hypothesis. What is really interesting, he says, is that most plants do not have different sexed organisms, but are hermaphrodites. “Perhaps this can help us derive some theory independent of sex determination in animals, because in *Arabidopsis* there is no such thing, so we have to think, what are the properties in common between animals and plants? Why do they have similar kinds of phenomenon?”

For their study Wu et al. searched the ProteinHistorian database for the ages of genes in *Arabidopsis thaliana*, a common model organism in plant biology and genetics. They looked for these genes in the microarray expression data of 79 tissue samples from *Arabidopsis* taken in different stages of development. Wu and his team found that mature pollen expresses more young genes than any other tested tissue (a finding consistent with other studies).

Interestingly, perhaps strangely, the team found that new genes are more likely to be expressed in the vegetative nucleus than in the sperm cells in a pollen grain. (Mature pollen is composed of three cells: One vegetative nucleus and two sperm cells.) The sperm cells are the parental cells in a pollen grain, contributing DNA to the next generation. The vegetative nucleus is important for fertilization as it helps grow the pollen tube to transport the male gamete cells.

“We expected that vegetative nucleus cells could not be the source of new genes if they do not contribute to the next generation of individuals,” says Wu. “Genes originating in these cells would be dead-ends, the same as if they duplicated in somatic cells of animals. […] It is paradoxical that new genes are more likely generated from the VN that does not contribute DNA directly to the progeny.”

For some plant geneticists, however, the phenomenon does not seem quite so strange. “If it helps the pollen grow and fertilize then it’ll be an advantage,” says Jeff Bennetzen, who studies the genome structure and evolution of plants at the University of Georgia. “I don’t find it as paradoxical as they do. Pollen growth and pollen fertilization is under as much selection as anything else.”

A lot of genes that are not expressed in other tissues are expressed in the vegetative nucleus, Bennetzen says. Many transposable elements, the origin of many sequences that look like genes tend to be expressed in high levels in the vegetative nucleus as well. “So it’s a good place to think that selection could first work on them,” says Bennetzen, “and if they have an advantage then they persist and eventually become real genes.”

Bennetzen would like to see what roles the assumed new genes play in plant development. The study, he says, is “provocative, a good starting point for investigating if theses ‘new genes’ have selectable function.”

The authors write that very few of the new genes are involved in the reproductive system, instead they seem to be slanted toward adaptive responses to environmental stimuli.

“There is a lot of bizarre phenomenon [in biology] challenging us to do further research,” says Long, delighted to have new questions to ponder. “When I was a grad student people who worked on plants, animals, bacteria rarely talked to each other. Today there are more and more connections identified, it helps us understand life as a total and complete unit, a whole connected one.”
